# Network Analysis of Pathogenesis Markers in Murine Chagas Disease Under Antimicrobial Treatment

**DOI:** 10.3390/microorganisms12112332

**Published:** 2024-11-15

**Authors:** Nayra Dias, Marina Dias, Andressa Ribeiro, Nélio Gomes, Aline Moraes, Moisés Wesley, Carlito Gonzaga, Doralina do Amaral Rabello Ramos, Shélida Braz, Bruno Dallago, Juliana Lott de Carvalho, Luciana Hagström, Nadjar Nitz, Mariana Hecht

**Affiliations:** 1 Interdisciplinary Laboratory of Biosciences, Department of Pathology, Faculty of Medicine, University of Brasília, Brasília 70910-900, Brazil; nayragda@gmail.com (N.D.); marina98dias@gmail.com (M.D.); andria.ribeiro@gmail.com (A.R.); neliogmoura@gmail.com (N.G.); alinesilvamoraes_df@yahoo.com.br (A.M.); professormoisesw@gmail.com (M.W.); carlitogcj@gmail.com (C.G.); dorarabello@gmail.com (D.d.A.R.R.); dallago@unb.br (B.D.); julianalott@gmail.com (J.L.d.C.); loubex@hotmail.com (L.H.); nadjarnitz@gmail.com (N.N.); 2 Institute of Exact and Technological Sciences, Federal University of Amazonas, Manaus 69000-000, Brazil; shelidabraz@gmail.com

**Keywords:** Chagas disease, *Trypanosoma cruzi* strains, gut microbiota, antibiotics, immune response, parasite burden, tissue inflammation, correlation analysis

## Abstract

Chagas disease (CD), a disease affecting millions globally, remains shrouded in scientific uncertainty, particularly regarding the role of the intestinal microbiota in disease progression. This study investigates the effects of antibiotic-induced microbiota depletion on parasite burden, immune responses, and clinical outcomes in BALB/c mice infected with either the *Trypanosoma cruzi* Colombiana or CL Brener strains. Mice were treated with a broad-spectrum antibiotic cocktail before infection, and parasite burden was quantified via qPCR at 30 and 100 days post-infection (dpi). Immune responses were analyzed using flow cytometry and ELISA, while histopathology was conducted on cardiac and intestinal tissues. Antibiotic treatment uncovered strain-specific correlations, with Colombiana infections affecting *Bifidobacterium* populations and CL Brener infections linked to *Lactobacillus.* Microbiota depletion initially reduced parasite burden in the heart and intestine, but an increase was observed in the chronic phase, except in the CL Brener-infected gut, where an early burden spike was followed by a decline. Antibiotic-induced bacterial shifts, such as reductions in *Bacteroides* and *Bifidobacterium*, promoted a more pro-inflammatory immune profile. These findings highlight the importance of microbiota and strain-specific factors in CD and suggest further research into microbiota manipulation as a potential therapeutic strategy.

## 1. Introduction

Chagas disease (CD) is a neglected tropical disease that affects approximately eight million individuals globally and results in nearly 12,000 deaths each year [1]. Its etiological agent, *Trypanosoma cruzi* (Tc), is a heteroxenic, flagellated protozoan primarily transmitted through triatomine insects [1]. The disease progresses from an acute phase, often asymptomatic but characterized by high parasitemia, to a chronic phase in which parasite load is significantly reduced. However, this chronic phase marks the onset of cardiac and digestive impairment in approximately one-third of patients, potentially leading to severe, life-threatening complications [2]. 

The pathogenesis of CD is complex and remains incompletely understood, involving multifactorial host–parasite interactions that drive tissue damage and disease progression [3]. Key among parasite-related factors is the genetic diversity within Tc, which comprises six discrete typing units (DTUs; TcI through TcVI), each with distinct genetic, ecological, and pathogenic profiles that shape CD’s epidemiology and clinical trajectory [4]. For instance, TcI is predominantly associated with sylvatic transmission cycles and is frequently linked to milder or indeterminate clinical forms, whereas TcII exhibits a strong association with severe cardiac manifestations in humans [4,5]. Hybrid DTUs, such as TcVI, which arose from recombination events between strains, are commonly implicated in domestic transmission and correlate with both cardiac and digestive forms of CD, illustrating the role of genetic variation of the parasite in influencing clinical outcomes [6,7]. 

Host-related factors, particularly immune responses, significantly influence susceptibility to infection and the severity of CD manifestations [8]. Emerging research underscores the potential role of the intestinal microbiota in modulating CD progression, aligning with findings from other infectious diseases where microbiota homeostasis is known to shape immune function [9,10]. Microbial dysbiosis, in particular, has been linked to immune dysregulation, which may exacerbate disease severity by impairing the host’s ability to control parasitic infection effectively. 

Germ-free (GF) mice with no gut microbiota experienced worsened Tc infections, likely due to reduced levels of immune mediators like IFN-γ, TNF-α, nitric oxide, and anti-parasite antibodies [11,12]. Colonizing GF mice with specific bacteria, such *as Escherichia coli* and *Peptostreptococcus*, improved survival, indicating that certain microbiota components can alleviate infection severity by modulating immunity [13]. However, other bacteria like *Bacteroides fragilis* worsened outcomes, potentially by driving a Th2-skewed response that favors parasite persistence [14,15]. Tc infection in mice also alters the gut microbiome and metabolome, affecting fatty and bile acid pathways, which may enhance parasite survival by promoting anti-inflammatory responses [16]. Similar shifts in microbial profiles have been noted in infected humans, suggesting Tc may be linked to specific microbiota profiles that support persistence and immune modulation [17]. 

Building on these findings, this study aims to further elucidate the role of the gut microbiota in CD pathogenesis by assessing the impact of antibiotic-induced microbiota depletion on parasite burden, immune response, and clinical outcomes in murine models infected with the Tc Colombiana (DTU I) and CL Brener (DTU VI) strains. These strains were selected for their distinct genetic profiles and relevance to CD progression, providing an opportunity to explore strain-specific interactions in an antibiotic-altered microbiota environment. This approach may yield broader insights into microbiota contributions to CD pathogenesis and inform microbiome-targeted therapeutic strategies.

## 2. Materials and Methods

### 2.1. Experimental Groups, Antibiotic Administration, and Infection

**Figure 1:**
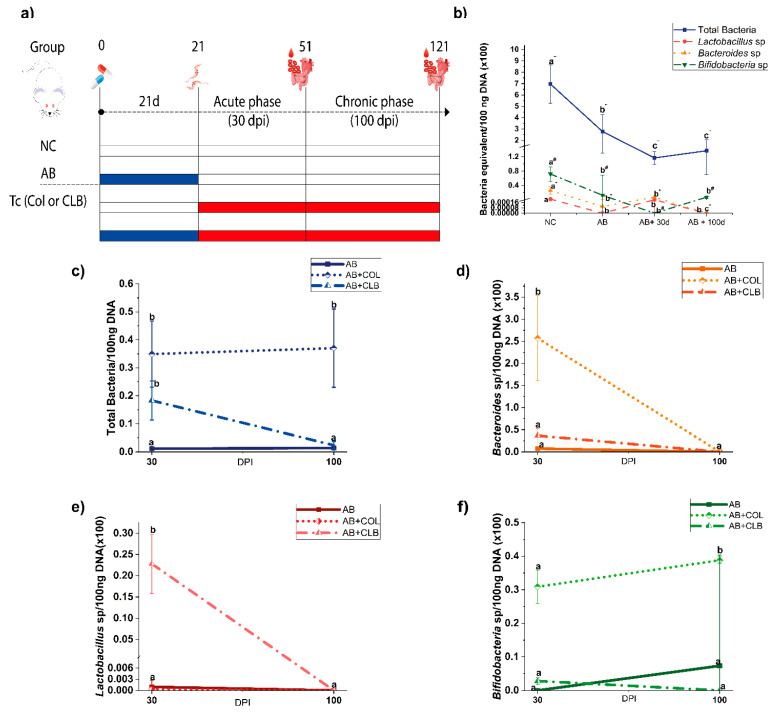
Antibiotic cocktail effectiveness and impact of *Trypanosoma cruzi* infection on the gut microbiome of treated mice. (**a**) Experimental groups. To evaluate the impact of antibiotic treatment on Chagas disease, mice were administered an antibiotic cocktail for 21 days (blue). Subsequently, the mice were infected with either the Colombiana or CL Brener strain of *T. cruzi* (red) and monitored for an additional 30 days (acute phase) or 100 days (chronic phase). Blood, cardiac, and large intestinal tissues were collected on days 21, 51, and 121. (**b**) Absolute quantification of total bacteria, *Lactobacillus* sp., *Bacteroides* sp., and *Bifidobacterium* sp. populations in non-infected, antibiotic-treated mice. (**c**–**f**) Quantitative assessment of bacterial populations in *T. cruzi-*infected, antibiotic-treated mice: (**c**) total bacteria; (**d**) *Lactobacillus Bacteroides* sp., (**e**) *Lactobacillus* sp.; and (**f**) *Bifidobacterium* sp. NC: negative control (untreated and uninfected). AB: animals that received the antibiotic cocktail. COL: *T. cruzi* Colombiana strain. CLB: *T. cruzi* CL Brener strain. Distinct letter designations indicate statistically significant differences within the respective group (*p* ≤ 0.05). To facilitate the identification of the specific group each letter refers to, different superscript symbols were used in panel (**b**).

In this study, we used 60 female BALB/c mice, randomly divided into 12 experimental groups of 5 animals each (Figure 1a). Mice were obtained from the animal facility of the University of Brasília Medical School and maintained in a temperature-controlled environment under a 12/12 h light/dark cycle with ad libitum access to food and water. All experimental procedures were conducted in accordance with the institutional guidelines for the ethical care and use of laboratory animals, and the study was approved by the University of Brasília Animal Research Ethics Committee (CEUA) under protocol number 129/2019.

To drastically reduce the microbial diversity of mice gut, we treated them with a broad-spectrum antibiotic cocktail comprising neomycin (0.5 mg/mL, Sigma-Aldrich^®^, Saint Louis, MO, USA), metronidazole (0.5 mg/mL, Sigma-Aldrich^®^), ampicillin (0.5 mg/mL, Teuto^®^, Bielefeld, Germany), gentamicin (0.5 mg/mL, Novafarma^®^, Guarulhos, Brazil), and vancomycin (0.25 mg/mL, Teuto^®^) for 21 days prior to infection with Tc. The antibiotics in the cocktail target a range of bacterial groups: vancomycin primarily targets Gram-positive bacteria, ampicillin has a broad-spectrum effect against both Gram-positive and Gram-negative cocci, neomycin reduces ammonia-producing bacteria in the gastrointestinal tract, metronidazole acts against anaerobes, and gentamicin is effective against Gram-negative bacteria. To prevent sedimentation, each antibiotic was prepared individually once a week and stored in autoclaved glass bottles. The antibiotic cocktail was then prepared and administered to mice via their drinking water. Every two days, the drinking water bottles were sanitized and refilled with fresh antibiotic solution to maintain consistent dosing throughout the 21-day treatment period. The selected antibiotic concentrations were based on a prior study using this same combination, which effectively and significantly depleted the intestinal microbiota [18].

Tc and L6 cultures were obtained according to [3]. All female mice were infected with 1 × 10^5^ intraperitoneal injections of trypomastigote forms of Tc from Colombiana (DTU I) or CL Brener (DTU VI) strains. At 7 days post-infection (dpi), blood parasitemia was assessed by direct parasite observation under a 40× objective using an Olympus BX51 microscope (Olympus^®^, Victoria, Australia). If no parasitemia was detected, the procedure was repeated daily until the parasite was confirmed. Infection was verified in all animals up to the 14th day post-infection.

### 2.2. Sample Collection

At the designated endpoints of 30 and 100 dpi, Tc-infected mice were euthanized. Approximately 150 µL of blood was collected via cardiac puncture for serum extraction. Additionally, cardiac and large intestinal tissues were harvested for DNA isolation and subsequent histological analysis. In the cohort of treated but uninfected animals, intestinal tissues were collected after 21 days of antibiotic use to evaluate the treatment’s efficacy.

### 2.3. DNA Isolation and Quantitative PCR

The DNA was extracted from the tissue samples using the Wizard Genomic DNA Purification kit (Promega^®^, Madison, WI, USA), following the manufacturer’s instructions. There was no attempt to separate the mouse tissue from the bacterial component in order to avoid selecting bacteria closely associated with the murine tissue. Quantitative Polymerase Chain Reaction (qPCR) was employed to quantify Tc nuclear DNA (nDNA) and bacterial DNA in the test samples. Standard curves were generated for each target organism using serial dilutions (10^5^ to 10^−2^) of their respective DNA (i.e., Tc, *Bacteroides fragilis*, *Lactobacillus casei*, *Bifidobacterium longum,* and total bacteria).

The primer pairs used for detecting Tc nDNA (forward: 5′-GCAGTCGGCKGATCGTTTTCG-3′ and reverse: 5′-TTCAGRGTTGTTTGGTGTCCAGTG-3; Ref. [19]) exhibited an efficiency of 90.56%. The amplification conditions were as follows: 50 °C for 2 min, 95 °C for 10 min, and 40 cycles of 65 °C for 15 s, and 72 °C for 10 s.

For quantification of total bacteria, the primer pairs used exhibited an efficiency of 90.67%. The DNA was amplified using the primers forward: 5′-ATGGCTGTCGTCAGCT-3′ and reverse: 5′-ACGFGGCGGTGTGTAC-3′ 16sRNA [20]. The amplification conditions were: 50 °C for 2 min, 95 °C for 10 min, and 40 cycles of 55 °C for 30 s, and 72 °C for 10 s.

For quantification of *Bacteroides* sp., the primer pairs used exhibited an efficiency of 104.97%. The DNA was amplified using the primers forward: 5′-AGTAACACGTATCCAACCTG-3′ and reverse: 5′-GAC CAA TATTCC TCA CTG CT-3′ 16sRNA [20]. The amplification conditions were as follows: 50 °C for 2 min, 95 °C for 10 min, and 40 cycles of 61 °C for 60 s, and 72 °C for 10 s.

For *Lactobacillus* sp., the primer pairs used for detecting this genus of bacteria’s DNA exhibited an efficiency of 96.37% (forward: 5′-TGGAAACAGATGCTAATACCG-3′ and reverse: 5′-GTCCAT TGTGGAAGATTCCC-3′ 16sRNA) [20]. The amplification conditions were as follows: 50 °C for 2 min, 95 °C for 10 min, and 40 cycles of 61 °C for 20 s, and 72 °C for 10 s.

Finally, for quantification of *Bifidobacterium* sp., the primer pairs used exhibited an efficiency of 99.17% (forward: 5′-AGGGTTCGATTCTGGCTCAG-3′ and reverse: 5′-CATCCGGCATTACCACCC-3′ 16sRNA) [20]. The amplification conditions were as follows: 50 °C for 2 min, 95 °C for 10 min, and 40 cycles of 66 °C for 30 s, and 72 °C for 10 s, using 0.3 µL of primer.

Each qPCR plate included blank (DNA-free water), negative control, and positive control samples (DNA from uninfected and infected mice, respectively) to validate the results. To ensure consistency across plates, two points on the curve were included in each plate as calibrators. A correction factor between runs was applied to account for any differences in measurements, following the method described by [21].

### 2.4. Cytokines Measurement by Flow Cytometry

Interleukin-2 (IL-2), interleukin-4 (IL-4), interleukin-10 (IL-10), interleukin-6 (IL-6), interleukin-17 (IL-17A), tumor necrosis factor (TNF), and interferon-γ (IFN-γ) were measured with the Cytometric Bead Array (CBA) Mouse Th1/Th2/Th17 Cytokine Kit (BD), following the manufacturer’s instructions. The standard curve for each cytokine was generated using serial dilutions of the top standard supplied with the kit, spanning concentrations from 1:2 to 1:256. An LSRFortessa™ BD cytometer was used for data acquisition, and FCAP 3.0 software (BD Biosciences^®^, San Jose, CA, USA) was used for data analysis.

### 2.5. Indirect Enzyme-Linked Immunosorbent Assay (ELISA)

Serum samples were analyzed for parasite-specific and autoreactive antibodies using ELISA. IgM levels were quantified during the acute phase, while IgG levels were assessed during the chronic phase, as described by Wesley et al. (2019) [3]. In brief, ELISA plates were coated with 50 μL per well of parasite, cardiac, or intestinal antigens. For the heart and intestine, the antigen corresponded to an amount of 0.4 µg per well, while 0.75 µg of Tc antigen was used. Serum samples were diluted 1:100 in 2% milk-PBS and applied to the plates. Secondary antibodies (anti-mouse IgM or IgG; Sigma-Aldrich^®^) conjugated to alkaline phosphatase were used at a 1:1000 dilution. The reaction was developed using p-nitrophenyl phosphate (pnPP) substrate, and optical density (OD) readings were obtained at 450 nm with a BioTek GENE 5^®^ spectrophotometer (Agilent, Santa Clara, CA, USA). Cut-off values were determined for each antigen as the mean plus three times the standard deviation (MEAN + 3 × SD) of 10 negative controls. Samples with ODs exceeding 10% above the cut-off were considered positive, while those with ODs 10% below the cut-off were classified as negative.

### 2.6. Histological Analysis

Cardiac and large intestinal tissues were harvested at predetermined time points, fixed, and embedded in paraffin. Subsequently, 5 μm-thick sections were prepared from each tissue and stained with hematoxylin and eosin (H&E). Histopathological examination was conducted by two independent pathologists using an Olympus BX51 U-LH100HG optical microscope (Olympus Corporation, Tokyo, Japan). Tissue alterations were categorized according to the criteria established by [3]. Briefly, a semi-quantitative scoring system was employed to classify the intensity of myocardial inflammation, ranging from 0 (normal) to 4 (severe myocarditis), based on increased leukocyte infiltration within the tissue. The mean number of inflammatory cells per histopathological slide was calculated across 15 randomly selected fields, and subsequently categorized into severity levels based on predefined thresholds. Tissue hemorrhage and necrosis were also annotated.

### 2.7. Statistical Analysis

The Shapiro–Wilk test was used to assess the normality of the variables. Normally distributed variables were subjected to analysis of variance (PROC GLM), followed by Tukey’s multiple comparison test at a 5% significance level. Pearson correlation analysis (PROC PRINCOMP) was performed to examine the relationships among the measured variables, and the figure was generated using the R core package (version 4.4.1) [22,23]. Principal component analysis was conducted on the total raw data using the PROC CORR procedure. All statistical analyses were performed using the SAS^®^ program (version 9.3, Cary, NC, USA) at a 5% significance level. Data are presented as means and standard deviation.

## 3. Results

### 3.1. The Antibiotic Cocktail Effectively Induces a Long-Lasting Reduction in the Intestinal Microbiota

To elucidate the impact of the intestinal microbiota on the modulation of CD, our study employed a comprehensive antibiotic cocktail. Notably, a shift in fecal consistency from solidity to pastiness was observed after a 21-day course of antimicrobial treatment, indicative of alterations in the intestinal microbiota. The qPCR analysis revealed a significant decrease (*p* < 0.0001) in total bacterial abundance shortly after cessation of antibiotic use (21 days), which persisted for up to 100 days post-treatment cessation (Figure 1b). Moreover, the assessment of three pro-healthy bacterial genera, namely *Lactobacillus* sp., *Bacteroides* sp., and *Bifidobacterium* sp., demonstrated a discernible reduction in their populations throughout the study period among mice subjected to the antibiotic cocktail (*p* < 0.0001).

### 3.2. Impact of Trypanosoma cruzi Infection on the Gut Microbiome in Antibiotic-Treated Mice

Upon completion of the treatment regimen, the animals were inoculated with trypomastigote forms of either Colombiana or CL Brener strains of Tc. Quantitative analysis of the total bacterial load revealed that infection with the Colombiana strain resulted in a more than 27-fold increase in bacterial population in animals previously treated with antibiotics, observed during both the acute and chronic phases of infection. The CL Brener strain induced a significant 15-fold increase in bacterial load during the acute phase, but this increase was not observed during the chronic phase (Figure 1c).

When examining specific pro-health bacterial genera (Figure 1d–f), distinct patterns emerged between the Colombiana and CL Brener strains. At 30 dpi, Colombiana significantly increased the quantity of *Bacteroides* sp. (*p* = 0.0271) and *Bifidobacterium* sp. (*p* = 0.0001), whereas CL Brener led to an increase in the population of *Lactobacillus* sp. (*p* < 0.0001). Furthermore, at 100 dpi, infection with Colombiana in treated animals resulted in a significant increase (*p* = 0.0001) in *Bifidobacterium* sp.

### 3.3. Strain-Dependent Alterations in Parasite Burden Following Intestinal Microbiota Depletion

**Figure 2:**
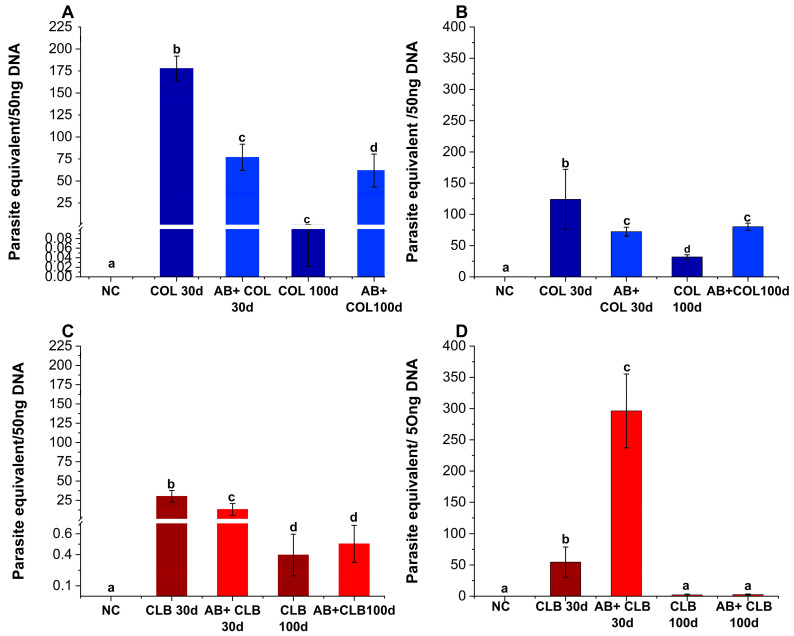
Microbiota-driven antibiotic strategy impacts *Trypanosoma cruzi* load. Quantification of parasite burden in cardiac tissue (**A**,**C**) and intestinal tissue (**B**,**D**) of mice subjected to 21 days of antibiotic cocktail treatment and subsequently infected with *T. cruzi* Colombiana strain (COL, blue) or CL Brener strain (CLB, red). The assessment was conducted at both 30 dpi and 100 dpi. The following groups were analyzed: NC: Negative control group (untreated, uninfected mice); COL30d: *T. cruzi* Colombiana strain-infected mice at 30 dpi; AB + COL30d: Antibiotic-treated and *T. cruzi* Colombiana strain-infected mice at 30 dpi; COL100d: *T. cruzi* Colombiana strain-infected mice at 100 dpi; AB + COL100d: Antibiotic-treated and *T. cruzi* Colombiana strain-infected mice at 100 dpi; CLB30d: *T. cruzi* CL Brener strain-infected mice at 30 dpi; AB + CLB30d: Antibiotic-treated and *T. cruzi* CL Brener strain-infected mice at 30 dpi; CLB100d: *T. cruzi* CL Brener strain-infected mice at 100 dpi; AB + CLB100d: Antibiotic-treated and *T. cruzi* CL Brener strain-infected mice at 100 dpi. Distinct alphabetical letters indicate statistically significant differences among groups (*p* < 0.0001).

The assessment of Tc parasite load within the cardiac tissue revealed an initial reduction of nearly 60% in parasitic burden among animals infected with the Colombiana strain, as facilitated by antibiotic administration (*p* < 0.0001; Figure 2A). Nevertheless, the observed parasitemia reduction was transitory, as parasite burden rebounded to levels exceeding that of untreated infected animals by a factor of 200 at 100 dpi in animals subjected to antibiotic cocktail therapy (*p* < 0.0001). A parallel trend was observed when analyzing parasite quantification within the animals’ intestinal tissue (Figure 2B), showing an initial reduction of approximately 30% in the parasitic load, followed by a 2.5-fold increase at 100 dpi (*p* < 0.0001). Remarkably, antibiotic cocktail treatment resulted in a sustained parasite burden throughout both the acute and chronic phases of infection.

In the setting of groups infected with the CL Brener strain, our findings indicate that, at 30 dpi, the application of antimicrobial therapy resulted in a substantial threefold reduction in parasite burden in the cardiac tissue (Figure 2C; *p* < 0.0001), concomitant with a sixfold increase in parasite abundance within the intestinal tissue (Figure 2D; *p* < 0.0001). Nevertheless, by 100 dpi, the effects of the treatment appeared to have waned, as they no longer exerted any discernible influence on the parasite load within any of the assessed tissues.

### 3.4. Antibiotic Treatment Has a Limited Impact on Immune Response to Trypanosoma cruzi

**Figure 3:**
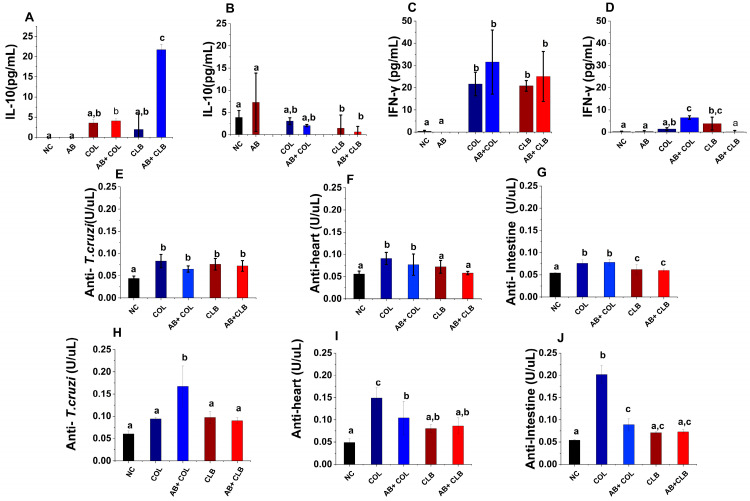
Immune response in *Trypanosoma cruzi*-infected mice treated with antimicrobials. Flow cytometry was employed to quantify (**A**) IL-10 at 30 dpi, (**B**) IFN-γ at 30 dpi, (**C**) IL-10 at 100 dpi, and (**D**) IFN-γ at 100 dpi within distinct experimental groups. Specific antibody titers against *T. cruzi* and cardiac and intestinal autoantibodies were determined at (**E**–**G**) 30 dpi and (**H**–**J**) 100 dpi. NC: Negative control group (untreated, uninfected mice); AB: Antibiotic-treated group; COL30d: *T. cruzi* Colombiana strain-infected mice at 30 dpi; AB + COL30d: Antibiotic-treated and *T. cruzi* Colombiana strain-infected mice at 30 dpi; COL100d: *T. cruzi* Colombiana strain-infected mice at 100 dpi; AB + COL100d: Antibiotic-treated and *T. cruzi* Colombiana strain-infected mice at 100 dpi; CLB30d: *T. cruzi* CL Brener strain-infected mice at 30 dpi; AB + CLB30d: Antibiotic-treated and *T. cruzi* CL Brener strain-infected mice at 30 dpi; CLB100d: *T. cruzi* CL Brener strain-infected mice at 100 dpi; AB + CLB100d: Antibiotic-treated and *T. cruzi* CL Brener strain-infected mice at 100 dpi. Distinct alphabetical letters indicate statistically significant differences among groups (*p* < 0005).

In the subsequent experimental phase, we evaluated the activation of the immune response to Tc in animals subjected to antibiotic therapy. Contrary to expectations, microbiota modulation exerted a minimal influence on both innate and adaptive immunity in infected mice. The production of Th1, Th2, and Th17 cytokines in treated and infected mice remained consistent with those observed in solely infected controls, irrespective of the Tc strain employed. Statistically significant discrepancies were exclusively identified in IL-10 levels at 30 dpi and IFN-γ levels at 100 dpi (Figure 3A–D). At 30 dpi, animals administered the antibiotic cocktail and infected with the CL Brener strain exhibited a tenfold elevation in IL-10 production compared to the infection-only group (*p* < 0.0001). However, this alteration was not evident in mice infected with the Colombiana strain. Concerning IFN-γ at 100 dpi, antibiotic treatment augmented cytokine production in animals infected with the Colombiana strain, whereas a more than 80-fold reduction was observed in mice infected with the CL Brener strain.

With respect to the adaptive immune response, antibiotic administration did not impact antibody titers against the parasite or autoantibodies targeting cardiac and intestinal tissues during the acute phase (Figure 3E–G). During the chronic infection phase (Figure 3H–J), outcomes varied contingent on the Tc strain. Specifically, microbiota depletion led to a twofold increase in anti-Tc antibody production in mice infected with the Colombiana strain (*p* < 0.0006), while antibodies against cardiac tissue were diminished by over 50% (*p* < 0.05). In animals treated with antibiotics and infected with the CL Brener strain, no substantial modifications were detected at 100 dpi.

### 3.5. Effect of Antibiotic Treatment on Cardiac and Intestinal Inflammation in Experimental Chagas Disease

To further assess the impact of antibiotic therapy on tissue integrity in infected mice, we examined the presence of amastigote nests and the severity of inflammatory infiltration, hemorrhage, and necrosis within cardiac and large intestinal tissues. Despite qPCR confirmation of Tc infection in both tissues, no amastigote nests were detected in either organ for animals infected with either parasite strain, regardless of antibiotic treatment.

**Figure 4:**
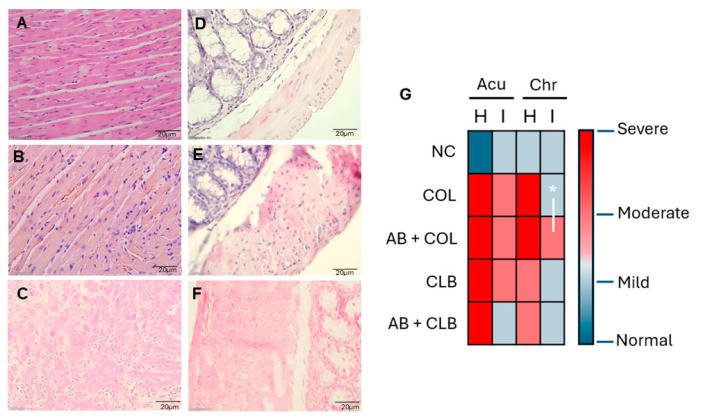
Inflammatory response in *Trypanosoma cruzi*-infected mice following antibiotic treatment. Hematoxylin and eosin (H&E)-stained histological sections reveal the normal cardiac and intestinal architecture in uninfected, untreated control mice (**A,D**). In *T. cruzi*-infected antibiotic-treated mice, representative images demonstrate: (**B**) moderate inflammation with diffuse and focal inflammatory infiltrates in the heart; (**C**) severe diffuse inflammatory infiltrates with associated parenchymal damage in the heart; (**E**) mild intestinal inflammation; and (**F**) moderate inflammation with parenchymal alterations in the intestine. (**G**) Leukocyte infiltration in cardiac and intestinal tissues was assessed during the acute (Acu) and chronic (Chr) phases of infection using a heatmap analysis. Inflammation severity was graded as follows: 0–0.2 (normal, dark blue), 0.3–1.0 (mild, light blue), 1.1–2.0 (moderate, light red), and 2.1–3.0 (severe, dark red). Scale bar represents 10 μm. NC: uninfected control group; COL: *T. cruzi* Colombiana strain; AB + COL: antibiotic-treated *T. cruzi* Colombiana strain; CLB: *T. cruzi* CL Brener strain; AB + CLB: antibiotic-treated *T. cruzi* CL Brener strain. H: heart. I: intestine. * *p* = 0.01.

Histological examination revealed focal and diffuse inflammatory infiltrates, ranging from mild to severe intensity. Focal necrosis and hemorrhage were found especially within the cardiac tissue of infected animals, as depicted in Figure 4A–F. Analysis of inflammatory infiltrates revealed a more pronounced reaction in cardiac tissue, compared to intestinal tissue during both acute and chronic infection phases (Figure 4G). Notably, the observed differences in inflammation pattern were primarily attributed to the infecting parasite strain rather than the antibiotic treatment regimen. Antibiotic administration elicited a significant increase in inflammation in intestinal tissue solely within the chronic phase of Colombiana strain infection (*p* = 0.01) compared to untreated ones.

### 3.6. Correlation and Principal Component Analysis of Antibiotic Impact on Chagas Disease

Isolated analysis of various pathogenesis markers in CD indicates minimal impact of antimicrobial therapy on several evaluated parameters. However, a complex model incorporating temporal correlations reveals distinct patterns and trends not apparent in univariate analysis.

**Figure 5:**
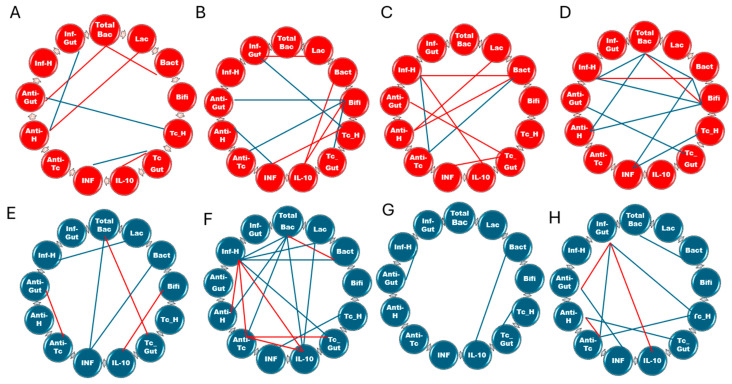
Pearson correlation network analysis of microbiota and host-associated parameters during *Trypanosoma cruzi* infection in antibiotic-treated mice. The interaction networks display correlation matrices for (**A**) untreated Colombiana-infected mice at 30 dpi, (**B**) Colombiana-infected mice treated with antibiotics at 30 dpi, (**C**) untreated Colombiana-infected mice at 100 dpi, and (**D**) Colombiana-infected mice treated with antibiotics at 100 dpi. Panels (**E**) through (**H**) present the corresponding correlation matrices for CL Brener-infected mice, with (**E**) representing untreated mice at 30 dpi, (**F**) CL mice treated with antibiotics at 30 dpi, (**G**) untreated infected mice at 100 dpi, and (**H**) CL Brener-infected mice treated with antibiotics at 100 dpi. TotalBac, total bacterial population; Lac, *Lactobacillus* sp.; Bact, *Bacteroides* sp.; Bif, *Bifidobacterium* sp.; Tc_H, cardiac parasite burden; Tc_Gut, intestinal parasite burden; IL-10, interleukin-10; IFN-γ, interferon-gamma; Anti-Tc, anti-*T. cruzi* antibody titers; Anti-H, anti-heart antibody titers; Anti-Gut, anti-intestinal antibody titers; Inf_H, cardiac inflammation; Inf_Gut, intestinal inflammation. The interaction network was made with correlations that presented significance level ≤ 0.05. Red lines: positive correlations. Blue lines: negative correlations.

In animals infected with the Colombiana strain at 30 dpi (Figure 5A,B), antibiotic administration resulted in new bacterial correlation patterns, suggesting an altered dysbiosis profile compared to non-treated infected animals. The modulation of *Bifidobacterium* sp. was particularly noteworthy, as it significantly impacted intestinal parasite burden, and the production of antibodies targeting both intestinal tissues and Tc. This bacterium, along with *Bacteroides* sp., exhibited a positive correlation with IL-10 levels.

Moreover, antimicrobial treatment led to a significant increase in the positive correlation between cardiac parasite load and IFN-γ levels. Conversely, a negative correlation between cardiac autoantibodies and IFN-γ levels was observed. Notably, the relationship between cardiac parasite load and intestinal autoantibodies, previously observed in untreated animals, was no longer evident. Instead, the cardiac parasite burden exhibited a direct influence on intestinal inflammation, which was also modulated by *Lactobacillus* abundance.

Upon progression to the chronic phase, antibiotic treatment strengthened existing correlations and introduced novel associations among analyzed parameters in Colombiana strain-infected animals (Figure 5C,D). *Bifidobacterium* sp. abundance continued to be a significant factor in the observed correlations among treated animals. This microorganism was associated with increased immune responses to cardiac antigens, leading to heightened cardiac inflammation. The overall bacterial load and *Bacteroides* abundance also contributed to these inflammatory processes. Notably, cardiac inflammation was no longer significantly correlated with IL-10 or anti-Tc antibodies. IFN-γ exhibited a negative correlation with *Bacteroides* and cardiac parasite burden, indicating that reduced IFN-γ levels were associated with elevated parasite loads in this organ.

In mice infected with the CL Brener strain, depletion of the gut microbiota markedly altered the dynamics of parasite–host interactions, rendering them significantly more complex during both the acute and chronic phases of infection. At 30 dpi (Figure 5E,F), antibiotic treatment clearly influenced cardiac inflammation by strengthening correlations with parasite load, anti-Tc and anti-heart antibody production, *Bacteroides, Lactobacillus*, and total bacterial counts. Furthermore, correlations involving IL-10 became statistically significant, with a decrease in the total bacterial count and *Lactobacillus* species abundance being associated with increased IL-10 levels. Additionally, IL-10 exhibited a positive correlation with anti-parasite antibodies, which were influenced by the intestinal parasite burden.

Analysis of CL Brener strain-infected animals at 100 dpi (Figure 5G,H) revealed unique correlation patterns compared to both untreated infected and Colombiana strain-treated groups. The bacterial populations examined did not exhibit significant correlations with disease markers. Nevertheless, novel interactions emerged. For instance, a decreased intestinal Tc burden was associated with an elevated titer of anti-cardiac antibodies, while the cardiac parasite load influenced the production of anti-Tc antibodies and intestinal inflammation. The latter was also dependent on the levels of IL-10, anti-Tc, and anti-gut antibodies.

**Figure 6:**
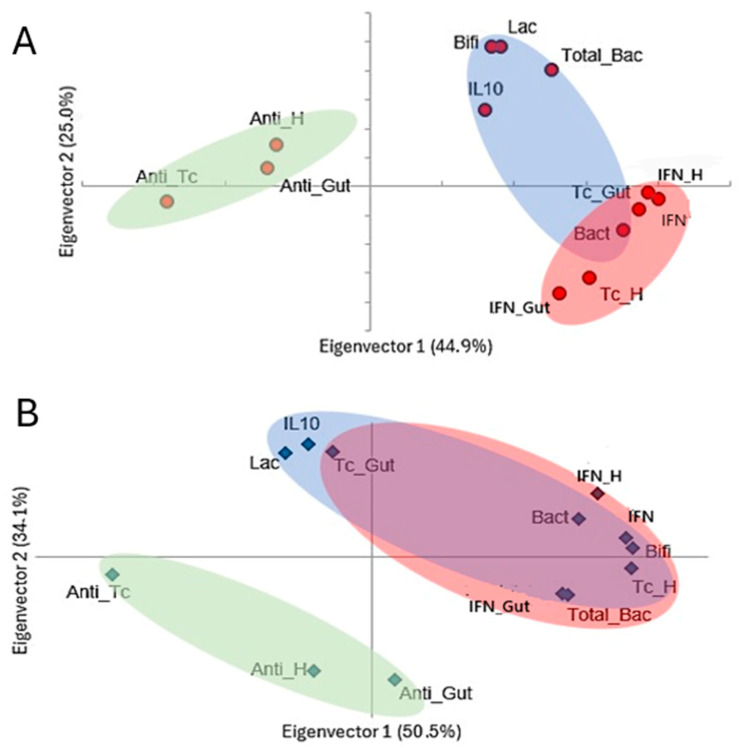
Principal component analysis of pathogenesis markers in a murine Chagas disease model under antibiotic treatment. (**A**) *T. cruzi*-infected mice. (**B**) *T. cruzi*-infected mice treated with antibiotic cocktail. The values in parentheses indicate the variance explained by each principal component (eigenvector). Similar elements grouped into clusters are highlighted by colored ellipses. TotalBac, total bacterial population; Lac, *Lactobacillus* sp.; Bact, *Bacteroides* sp.; Bif, *Bifidobacterium* sp.; Tc_H, cardiac parasite burden; Tc_Gut, intestinal parasite burden; IL-10, interleukin-10; IFN, interferon-gamma; Anti-Tc, anti-*T. cruzi* antibody titers; Anti-H, anti-heart antibody titers; Anti-Gut, anti-intestinal antibody titers; Inf_H, cardiac inflammation; Inf_Gut, intestinal inflammation.

Principal component analysis revealed key trends in variable behavior during disease progression in Tc-infected mice, both with and without antibiotic treatment (Figure 6). The first two principal components accounted for 69.9% and 84.6% of the observed variation in infected-only and treated-infected mice, respectively. In the untreated group, three distinct clusters emerged. The first cluster demonstrated a positive association between total bacteria, *Lactobacillus*, *Bifidobacterium*, and IL-10. The second cluster encompassed parasite burden in the heart and intestine, along with associated inflammation, and IFN-γ. *Bacteroides* were identified in the overlap region between the first and second clusters. The third cluster was in the opposite quadrant to the second and included antibodies against Tc and autoantibodies.

Upon microbiota depletion, these associations undergo substantial reconfiguration, with a notable overlap between the components of clusters 1 and 2. This reorganization allows for the observation of a closer association between intestinal parasitic load and *Lactobacillus* and IL-10, while total bacterial counts and *Bifidobacterium* integrate with the elements of cluster 2. Notably, antibodies remain distinct, forming an independent group within the third cluster.

## 4. Discussion

Over the years, the majority of studies on the pathogenesis of CD have predominantly focused on the direct host–parasite interaction [24,25], often overlooking the broader and more complex ecological context in which this interaction occurs. This context includes a diverse array of symbiotic and pathogenic organisms, particularly the intestinal microbiota, which play critical roles in health and disease processes. Given that the role of the intestinal microbiota in CD pathogenesis remains largely unexplored, this study utilized the disruption of the intestinal microbiota through antibiotic cocktails, demonstrating that such disruption can alter the correlations among the parasite, the host’s immune response, and tissue inflammation, with these effects being contingent upon the specific infecting strain.

It is important to highlight that the use of antibiotics as a tool to modulate the microbiota and subsequently influence the immune response has emerged as a compelling approach in models of infectious diseases [18,26]. This strategy complements other established methods of immune modulation, such as genetic engineering techniques that generate knockout (KO) or transgenic animal models. Although antibiotic-mediated modulation induces broader, less targeted changes to the microbiota, leading to indirect uncontrollable effects on both innate and adaptive immune responses [27,28], this approach is notably less labor-intensive and more cost-effective compared to the complexity involved in generating engineered models. Thereby, antibiotic treatment facilitates practical, short-term studies aimed at examining not only the immune response but also the multifaceted interactions between the pathogen, microbiota, and host. 

In our study, the antibiotic cocktail therapy resulted in a significant depletion of the gut microbiome, including commensal genera such as *Lactobacillus*, *Bacteroides*, and *Bifidobacterium*. These findings support the effectiveness of the protocol described by [18] in manipulating bacterial populations and demonstrate that antibiotic cocktails can be as effective in studies considering the effects of microbiota depletion [29]. The dysbiosis persisted well beyond antibiotic cessation, highlighting the enduring impact of antimicrobial agents on the gut microbial ecosystem. In fact, the combinatorial effect of multiple antibiotics may contribute to the prolonged disruption, as evidenced by [30], who observed a persistent reduction in human microbiome richness six months post-treatment with meropenem, gentamicin, and vancomycin. Moreover, the antibiotic spectrum can differentially affect microbial recovery, potentially leading to long-lasting alterations in the gut microbiota [31].

The antibiotic-mediated disruption of the microbiota significantly altered the relationships between markers of disease pathogenesis, as evidenced by principal component analysis, which was conducted without stratifying by strain or time. In untreated, infected animals, Tc load, IFN-γ, and *Bacteroides*—a bacterium implicated in both beneficial and detrimental inflammatory processes [32]—were clustered together, suggesting a synergistic role in inflammation. Conversely, total bacterial load, *Lactobacillus*, *Bifidobacterium*, and IL-10 were clustered, indicating an anti-inflammatory, homeostatic response. Antibodies targeting Tc and host antigens demonstrated a dual role, potentially contributing to both tissue repair and inflammation [24,33], highlighting the complex interplay between the immune response and disease progression.

Following antimicrobial treatment, alterations in total bacterial and *Bifidobacterium* counts led to their shift towards the pro-inflammatory cluster, implying a possible loss of their immunomodulatory and anti-inflammatory roles [32]. Moreover, the parasite load in the gut began to associate with anti-inflammatory elements, such as *Lactobacillus* and IL-10, potentially indicating that the parasite’s persistence in the intestine is less pathogenic than its dissemination to other tissues, such as the heart. Remarkably, antibodies continued to appear detached from both the pro- and anti-inflammatory axes, suggesting that microbiota depletion does not significantly alter their role in the pathogenesis of CD [34,35].

Further analysis of antibody production revealed that antibiotic treatment affected parasite-specific antibodies and autoantibodies only during the chronic phase, likely due to the local, non-systemic action of IgM antibodies secreted by intestinal mucosa-resident B lymphocytes in response to microbiota changes. In contrast, IgG antibodies are capable of translocating beyond the intestine to act in other tissues [36].

These findings provide a comprehensive overview of the effects of intestinal microbiota reduction in animals infected with Tc. However, the use of different Tc strains led to variations in immune responses and correlations across analyzed parameters, both with and without antibiotic intervention, as evidenced by distinct bacterial profiles between Colombiana and CL Brener. These strain-specific differences may be linked to the distinct protein composition and abundance of extracellular vesicles (EVs) secreted by each Tc strain [37], which could mediate specific interactions with the bacterial community. This hypothesis is supported by the role of the gastrointestinal tract as a major Tc reservoir, where both parasite- and host-derived EVs actively modulate the local infection environment—including the intestinal microbiome—thus influencing immune responses and disease progression [38].

Therefore, these strain-specific differences underscore the intricate interactions between the parasite, host immunity, and the intestinal microbiome, which are shaped by each Tc strain’s unique genetic and biochemical characteristics [5,39,40,41], with potential therapeutic implications that support a personalized approach to CD management.

The variability in these interactions, compounded by the diverse experimental models and the limited number of studies in this area [42], hindered direct comparisons of our results with previously reported findings. The subsequent sections will explore the specific effects of antimicrobial treatment on host–parasite dynamics for each of the Tc strains used in this study.

### 4.1. Colombiana Strain-Infected Mice

In animals treated and infected with the Colombiana strain, the total bacterial load remained stable throughout the experimental period. However, there was a significant reduction in the abundance of *Bacteroides* and *Lactobacillus* populations at 100 dpi, suggesting a compensatory increase in other bacterial genera. The phenomenon of bacterial population shifts following antibiotic administration is well-documented, often resulting in the proliferation of potentially pathogenic species [43,44].

In these animals, early antibiotic intervention led to a reduction in parasite burden within both the intestine and heart at 30 dpi. Our findings diverge from those reported by Vieira et al. and Duarte et al. [12,45], who observed that in mice infected with the Y strain, the loss of the natural microbiota either exacerbates or has no effect on parasite load. Notably, intestinal parasitism was inversely correlated with *Bifidobacterium* abundance, a genus previously implicated in anti-protozoal activity against *Giardia lamblia* and *Plasmodium berghei* [46,47]. Cardiac parasitism, on the other hand, was negatively correlated with IFN-γ production, a pro-inflammatory cytokine with known anti-Tc activity [34,48,49], in a model in which the presence of the parasite triggers INF-γ production, subsequently reducing parasite numbers [50].

In the long term (100 dpi), the microbiota modulated by antibiotic treatment led to parasite burdens that remained elevated at levels characteristic of the acute phase of infection, deviating from the typical disease progression [24]. In this context, the increased parasite load was associated with a lower production of INF-γ compared to levels observed during the acute phase, indicating that cytokine levels were insufficient to control parasite replication. Of note, it has been demonstrated that antibiotic-induced gut microbiota suppression can reduce systemic INF-I levels through the cGAS–STING–IFN-I pathway [51].

Concerning the humoral immune response, the significance of *Bifidobacterium* is again evident in the group of mice treated and infected at 30 dpi. In this group, a higher abundance of *Bifidobacterium* correlates with a reduced production of antibodies against Tc, as well as against intestinal antigens, and to a lesser extent, against cardiac antigens (*p* = 0.06). This observation aligns with the known ability of bifidobacteria to promote the development of a tolerogenic response to various antigens [52] likely facilitated by their positive association with the anti-inflammatory cytokine IL-10. Supporting this, it was demonstrated that surface components of *B. longum* can stimulate peripheral blood mononuclear cells to secrete IL-10 [53].

During the chronic phase, increased *Bifidobacterium* abundance correlated with decreased cardiac autoantibody titers and attenuated cardiac inflammation. Additionally, a reduction in *Bacteroides* populations was associated with ameliorated cardiac inflammation. These findings align with previous studies demonstrating the impact of gut microbiota composition on inflammatory processes in various organs beyond the intestine [54,55]. While these results may suggest a potential cardioprotective effect of antibiotic therapy in Colombiana strain-infected mice, long-term follow-up studies are warranted to confirm this hypothesis, particularly given the observed increase in parasite load in these animals, along with an inflammatory response comparable to that seen in non-treated infected positive controls.

Conversely, intestinal inflammation was exacerbated at 100 dpi in antibiotic-treated mice, coinciding with a reversal of the correlation between intestinal inflammation and anti-Tc antibody production compared to untreated infected controls. This observation suggests a potential role for molecular mimicry, whereby IgG antibodies raised against the parasite may cross-react with intestinal proteins [56,57]. This process could be further amplified by the increased levels of systemic INF-γ observed in these animals [58,59].

### 4.2. CL Brener Strain-Infected Mice

Antimicrobial treatment of mice infected with the Tc CL Brener strain had a limited impact on the overall bacterial composition compared to uninfected controls. The exception was the significant increase in *Lactobacillus* abundance at 30 dpi. Contrary to previous reports demonstrating the anti-inflammatory effects of this bacterial genus [60,61,62], our findings indicate that the expansion of *Lactobacillus* abundance in treated mice in acute CD is associated with decreased IL-10 production. This discrepancy may be attributed to the unique context of Tc infection, where a pro-inflammatory environment is crucial for combating the parasite [56,63]. The previous studies cited likely employed non-infected models, limiting their applicability to infectious disease settings.

The total bacterial load was a significant factor in the observed correlations with host immune responses. A decrease in total bacterial abundance was associated with exacerbated inflammation. Previous studies have demonstrated the crucial role of the intestinal microbiota in regulating NF-κB expression, a transcription factor involved in the activation of various inflammation-related genes and modulation of which is implicated in the development of several pathologies, including cardiovascular and autoimmune diseases [64].

In the context of combating Tc, previous antibiotic therapy in Tc-infected mice resulted in increased intestinal parasitism at 30 dpi. It is known that specific disruptions to the intestinal microbiota can promote alterations in linoleic acid metabolism, which may immunomodulate the host’s immune response to favor the persistence of the parasite within the host organism [65]. Notably, the elevated parasite burden was associated with increased levels of anti-Tc antibodies. These antibodies, along with cardiac autoantibodies, may contribute to the observed cardiac inflammation in the infected animals. However, given that this is an early stage of infection, it is plausible that autoreactivity may serve a protective rather than a detrimental role, as IgM autoantibodies have been implicated in the recovery of damaged tissue [66,67].

As CD progresses to its chronic phase and parasite burden naturally declines, antibiotic administration results in a negative correlation between cardiac parasite load and anti-Tc, as well as between intestinal parasite load and heart-specific autoantibodies. This altered immunological profile may contribute to ongoing autoreactive processes that are deleterious to the host’s cardiac tissue. However, no exacerbation of the inflammatory response was observed in the antibiotic-treated animals, possibly due to the relatively early stage of chronic infection at which the mice were assessed.

## 5. Conclusions

This study provides new insights into the complex interplay between the intestinal microbiota, Tc infection, and the host immune response in the context of CD pathogenesis. By disrupting the gut microbial ecosystem through antibiotic therapy, we demonstrated significant alterations on host–parasite dynamics, highlighting the strain-specific effects on microbial composition and immune modulation. Notably, the findings suggest that alterations in the gut microbiome have implications for both acute and chronic phases of infection. The differential effects observed between the Colombiana and CL Brener strains underscore the importance of considering the DTU diversity of Tc when evaluating therapeutic strategies.

Furthermore, while antibiotic treatment can lead to increases in parasite burden, it can also have unintended consequences, such as limited effects on the immune response and minimal development of inflammatory conditions. Thereby, the complex and sometimes contradictory effects of antibiotic-induced dysbiosis necessitate careful consideration of long-term consequences. Aiming to enhance future studies, experimental design should incorporate a comprehensive characterization of the intestinal microbiota across the different experimental groups via 16S rRNA sequencing, along with longitudinal assessment of each animal’s fecal microbiota throughout the study period. Likewise, examining the specific effects of individual antibiotics on microbiota modulation and their contributions to CD progression could yield essential insights into the pathogenesis of this condition.

Additionally, research should concentrate on elucidating the precise mechanisms by which specific alterations in the microbiota influence host immunity and disease outcomes. Considering the significant role of microbiota in host immune responses, our findings in the murine model may have important implications for human CD. While differences between murine and human microbiomes warrant careful interpretation, the observed effects of microbiota modulation on immune parameters and disease progression could suggest new therapeutic avenues. Investigating these mechanisms in human models may elucidate whether microbiota-targeted interventions, such as probiotics, prebiotics, or tailored antibiotic regimens, could serve as adjunctive strategies to improve disease outcomes in Chagas patients.

## Data Availability

The original contributions presented in the study are included in the article, further inquiries can be directed to the corresponding author.
